# Early Predictors of Enteric Fever in Children Presenting With Fever Without Focus: A Prospective Study

**DOI:** 10.7759/cureus.83907

**Published:** 2025-05-11

**Authors:** Priyanka Kala, Rekha Mittal, Shyam Kukreja, Laxmi Narayan Taneja, Sandeep Kala

**Affiliations:** 1 Pediatrics and Neonatology, Max Super Speciality Hospital, New Delhi, IND; 2 Pediatric Neurology, Max Super Speciality Hospital, New Delhi, IND; 3 Pediatric Infectious Diseases, Max Super Speciality Hospital, New Delhi, IND; 4 Pediatric Medicine, Max Super Speciality Hospital, New Delhi, IND; 5 Neurological Surgery, Max Super Speciality Hospital, New Delhi, IND

**Keywords:** clinical diagnosis, early predictors, enteric fever, salmonella, typhoid

## Abstract

Objective

Blood culture is the gold standard for diagnosing enteric fever (EF), but its unavailability in many health facilities often leads to the overuse of antimicrobials. This study aimed to determine whether EF can be predicted early using clinical features and basic laboratory parameters, thus bypassing the need for blood culture.

Methodology

This prospective cohort study, conducted from September 2015 to February 2017 in Delhi, included children (6 months to 15 years old) presenting with "fever without focus" within the first week of onset. Based on the investigation results, children were categorized into either Group I: blood culture positive for EF - *Salmonella* Typhi/Paratyphi; Group II: blood culture negative for EF but had another diagnosis; or Group III: cases in which the cause could not be established or were treated with empirical antibiotics, considered as the ambiguous group and excluded from the study.

Results

Out of the 350 cases, blood cultures confirmed EF in 73 children; 112 children had another diagnosis subsequently, and 165 cases could be placed in Group III. Blood culture is considered the gold standard. Logistic regression was applied to statistically significant clinical and laboratory parameters in children in Group I to identify independent predictors of EF. Duration of fever >4 days, rising trend of fever, coated tongue, splenomegaly, C-reactive protein (CRP) (>25 mg/L), serum glutamic pyruvic transaminase (SGPT) (>40 IU/L), and absolute eosinopenia were independent predictors of EF in this study.

Conclusion

In resource-limited settings, and short of blood culture, early prediction of EF is possible with clinical features and simple laboratory investigations.

## Introduction

Enteric fever (EF) is a potentially life-threatening acute febrile systemic infection, encompassing two types of salmonellosis: typhoid fever and paratyphoid fever. Typhoid is caused by *Salmonella enterica* serotype Typhi bacteria, also called *Salmonella* Typhi. Paratyphoid is caused by the bacterium *S. enterica* of serotype Paratyphi A, usually, and occasionally by Paratyphi B or Paratyphi C. Paratyphoid infections constitute about 20% of all cases of EF [1]. The gold standard for the diagnosis of EF is blood culture, the results of which are received after two to three days. However, this facility is not available in many health centers in our country, particularly in remote and peripheral areas. The other commonly used tests for the diagnosis of typhoid fever are the typhidot and Widal tests. Though typhidot is a rapid, easy, and affordable test to detect antibodies IgM and IgG against *S.* Typhi, its use is discouraged due to low sensitivity and specificity [2]. The Widal test, like all serological tests, measures the rise in antibody levels against *Salmonella* antigens O-somatic and H-flagellar, but this takes 7-14 days, which limits its applicability in early diagnosis [3]. Thus, there are no reliable tests to suspect typhoid fever while awaiting the blood culture result, and there is also limited literature on the usefulness of clinical features in the early diagnosis of EF.

## Materials and methods

This prospective cohort study was conducted in the Pediatrics Department of a 400-bed tertiary care hospital in New Delhi, India, from September 1, 2015, to February 28, 2017, after obtaining ethical clearance. All children aged 6 months to 15 years with "fever without focus" within the first week of fever were included in the study after obtaining written consent from the parent/guardian. Children presenting in both OPD and IPD were included. After documenting clinical details related to fever and associated features, investigations - including complete hemogram, C-reactive protein (CRP), urine analysis, serum glutamic pyruvic transaminase (SGPT), blood culture (Bactec^®^ System; Becton, Dickinson and Company, Franklin Lakes, NJ, USA), malaria antigen, and peripheral smear - were conducted in all cases to identify the cause of fever. Culture samples were taken from children with fever lasting three days or more, or earlier if they showed severe symptoms, such as a fever above 103°F, poor feeding, or lethargy. Additional investigations, including imaging and the Mantoux test, were conducted as needed based on clinical conditions.

Results were compiled after the seventh day of enrollment, and patients were sorted into three groups: (1) Group I: EF - children with positive blood cultures for *S.* Typhi or Paratyphi; (2) Group II: non-EF (NEF) - children with negative blood cultures for *S.* Typhi or Paratyphi, those with another obvious cause of fever, or those whose fever resolved without antibiotics; and (3) Group III: ambiguous - children with negative blood cultures for *S.* Typhi or Paratyphi, no identifiable cause of fever, and who were treated with empirical antibiotics. This group was excluded from statistical analysis.

Sample size

To determine the sample size, the primary endpoint was the percentage of culture-positive cases. A study by Farooqui et al. [4] reported that *Salmonella* accounted for 66% of blood cultures. With a 5% margin of error at a 95% confidence level, the minimum sample size needed was 345. We included 350 cases in this study. The sample size calculation formula is as follows: $n = \frac{Z^2_{\alpha/2} \cdot \pi (1 - \pi)}{L^2}$, where Z_α/2_ = 1.96, π = 0.66, and L = 0.05.

Statistical analysis

Statistical analysis was performed using IBM SPSS Statistics for Windows, Version 20 (Released 2011; IBM Corp., Armonk, NY, USA) to identify the frequency and significance of historical, clinical, and laboratory parameters of culture-positive EF cases. The Chi-square test was used for qualitative parameters, and the Student's t-test was used for quantitative data. Logistic regression was then conducted on statistically significant parameters, using blood culture as the gold standard. Sensitivity and specificity were also calculated.

## Results

Out of the 350 total cases, 73 were blood culture-positive EF (Group I), 112 were NEF (Group II), and 165 cases were ambiguous (Group III). This ambiguous group was excluded from analysis. The non-EF group included cases that turned out to be dengue, urinary tract infection (UTI), respiratory tract infection (RTI), chikungunya, etc. The mean ages were 6.36 (±4.39) years for Group I and 7.7 (±4.89) years for Group II. In Group I, 48% of cases were aged 1-5 years. Group I comprised 68% males and 32% females, while Group II had 66% males and 34% females. Although males outnumbered females in both groups, the difference in sex distribution was not statistically significant. In Group I, the highest number of cases occurred in November (12 cases), followed by April (11 cases). Group II saw the most cases from July to October. Seasonal distribution indicated that most EF patients presented around the monsoon season. Almost all patients in both EF and NEF belonged to the upper-middle class. The majority drank reverse osmosis (RO) water - 95.9% in EF and 97.3% in NEF - while very few used municipal water directly.

**Figure 1:**
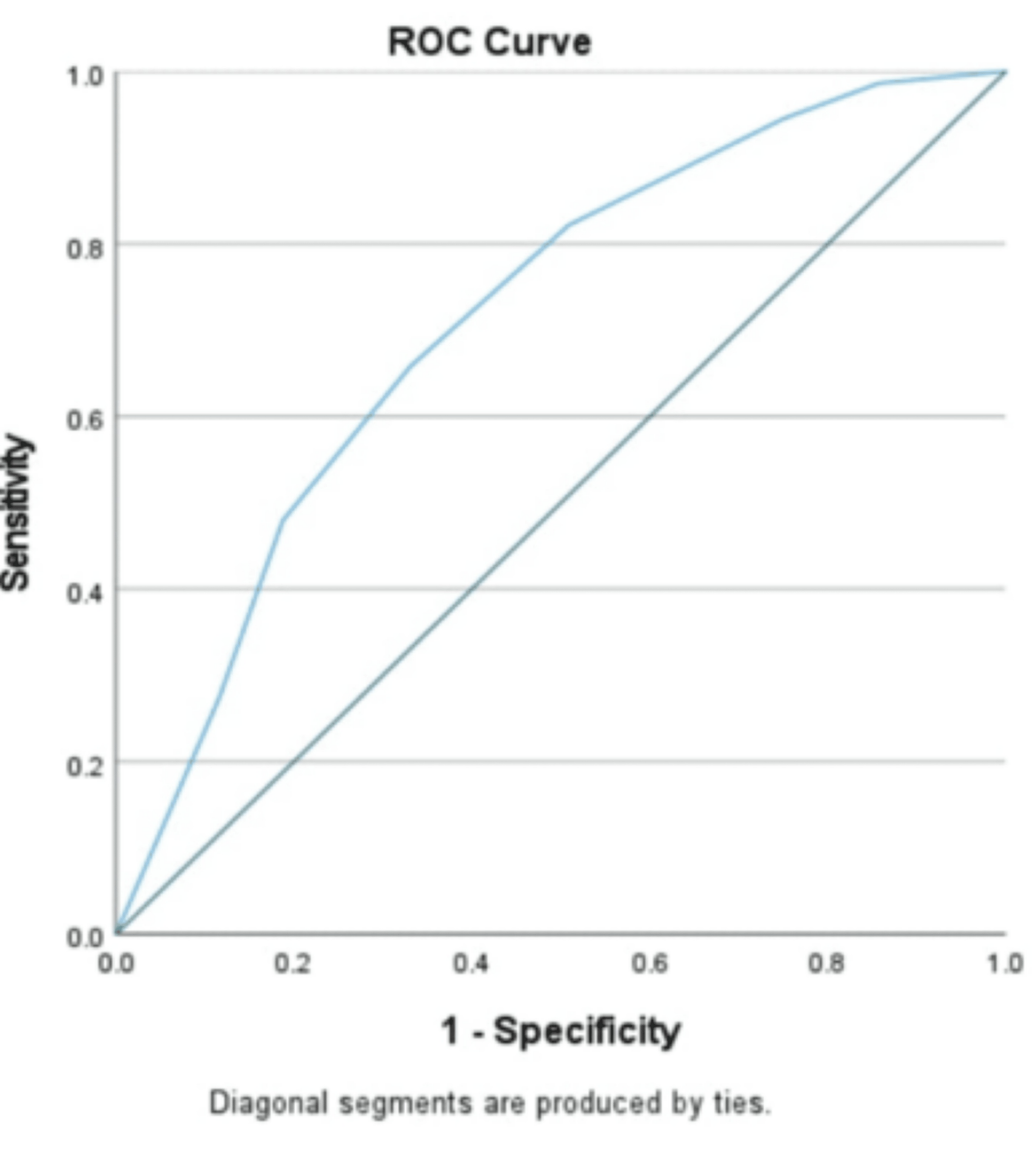
ROC curve for duration of fever ROC, receiver operating characteristic

Blood cultures were positive in 73 out of 350 enrolled cases, with 65 isolates identified as *S.* Typhi and 8 as *S.* Paratyphi A; no *S.* Paratyphi B or C were found. The mean temperature was 103.2°F in the EF group and 103.1°F in the NEF group. A higher proportion of EF patients had fever lasting more than four days, along with rising trends of fever (the fever temperature gradually increasing over time from low grade to higher grades) and loose stools - a difference that was statistically significant. The receiver operating characteristic (ROC) curve (Figure 1) indicated a best cut-off of 4.5 days for fever duration; thus, >4 days was used as a reliable predictor for EF.

The typhoid vaccine was received in 36 (64%) cases in Group I and 54 (59%) cases in Group II. There was no statistically significant difference found between the two groups. Most information was collected by verbal recall only from parents. In the absence of documentary evidence regarding the timing and nature of the vaccine, further analysis was not done. A family history of febrile illness was reported in 5.5% of EF cases and 4% of NEF cases. Upon applying logistic regression to significant parameters (i.e., duration of fever, rising trend of fever, and loose stools), the duration of fever >4 days and a rising trend of fever were found to be strong independent predictors of EF. Other symptoms, including abdominal pain, headache, appetite loss, constipation, rash, cough, and breathing difficulty, were not statistically significant.

Out of all clinical features, coated tongue, splenomegaly, and hepatomegaly were statistically significant for EF. However, logistic regression analysis revealed that only coated tongue and splenomegaly were strong independent predictors of EF. Splenomegaly with fever ≤4 days and blood culture positive for EF was seen in 12.3% of cases.

**Figure 2:**
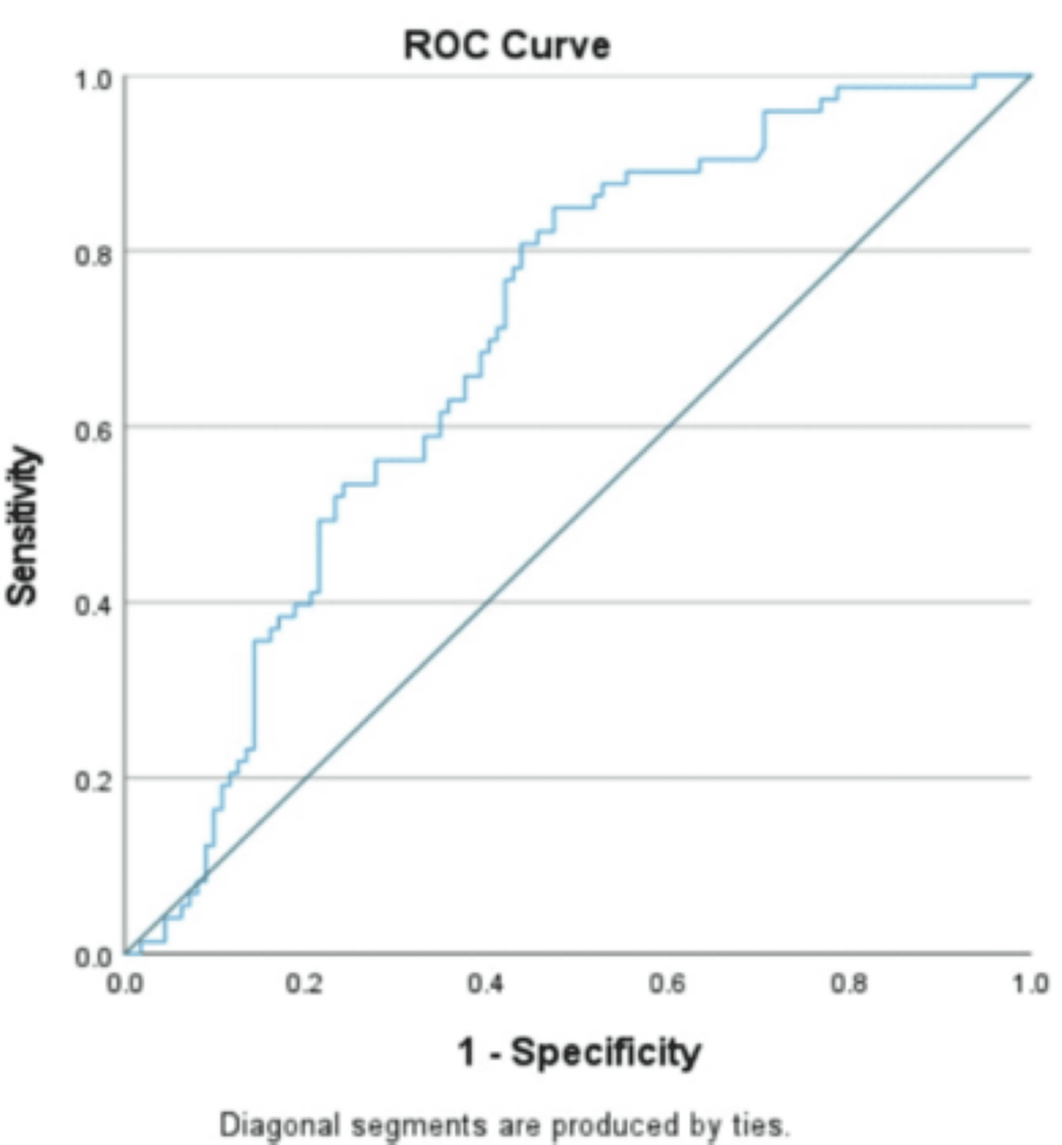
ROC curve for CRP ROC, receiver operating characteristic; CRP, C-reactive protein

Amongst the various laboratory parameters studied, normal total leucocyte count (TLC) counts (4000-11,000/mm³), relative neutrophilia (>50%), absolute eosinopenia (0%), CRP (>25 mg/l), normal platelet counts (1,50,000-4,00,000/mm³), and SGPT (>40 IU/L) were significantly seen in EF cases. It was also noted that EF in the age group <2 years showed leucocytosis in seven cases. Logistic regression identified eosinopenia, elevated CRP, and SGPT as strong independent predictors. The CRP cut-off was determined using the ROC curve (Figure 2). 

Table 1 summarizes all statistically significant parameters, while Table 2 presents the regression analysis of the most significant parameters. Table 3 shows the sensitivity and specificity of all significant parameters.

**Table 1:** Significant parameters for Group 1 (EF) including history, clinical, and laboratory parameters TLC, total leucocyte count; CRP, C-reactive protein; SGPT, serum glutamic pyruvic transaminase; EF, enteric fever

Parameters	Group I, N (%)	Group II, N (%)	Chi-square value (df)	t-value (df)	p-value	OR (95% CI)
Rising trend of fever	30 (41)	10 (9)	26.984 (1)	-	0.001	7.11 (3.19-15.82)
Loose stool	22 (30)	19 (17)	4.445 (1)	-	0.035	2.11 (1.04-4.26)
Coated tongue	39 (53.4)	18 (16)	28.926 (1)	-	0.001	5.99 (3.02-11.85)
Splenomegaly	31 (42.5)	8 (7)	33.144 (1)	-	0.001	9.59 (4.07-22.58)
Hepatomegaly	35 (48)	15 (13.4)	26.752 (1)	-	0.001	5.95 (2.92-12.13)
Duration of fever (>4 days)	48 (56.5)	37 (33)	-	31.190 (184)	0.001	0.26 (0.14-0.48)
TLC (4000-11,000/mm^3^)	61 (83.6)	56 (50)	-	20.409 (184)	0.001	4.54 (1.26-16.6)
Neutrophils (>50%)	64 (87.7)	84 (75)	-	57.638 (184)	0.035	2.37 (1.04-5.37)
Eosinophils (0%)	66 (90.4)	78 (69.6)	-	5.752 (184)	0.001	4.11 (1-12.9)
Platelet (1,50,000-4,00,000/mm^3^)	68 (93)	79 (71)	-	24.703 (184)	0.001	4.21 (1.03-12.2)
CRP (>25 mg/L)	60 (82.2)	52 (46.4)	-	10.951 (184)	0.001	4.75 (2.43-9.27)
SGPT (>40 IU/L)	42 (57.5)	27 (24.1)	-	3.366 (183)	0.001	4.26 (2.26-8.04)

**Table 2:** Logistic regression analysis for significant history, clinical, and laboratory parameters of Group 1 (EF) CRP, C-reactive protein; SGPT, serum glutamic pyruvic transaminase; EF, enteric fever

Parameters	p-value	OR (95% CI)
Duration of fever (>4 days)	0.046	2.26 (1.01-5.05)
Rising trend of fever	0.015	3.46 (1.26-9.43)
Coated tongue	0.007	3.36 (1.38-8.13)
Splenomegaly	0.001	7.24 (2.49-20.8)
CRP (>25 mg/L)	0.001	5.26 (2.18-12.6)
SGPT (>40 IU/L)	0.002	3.77 (1.63-8.69)
Eosinophils (0%)	0.015	3.17 (1.256-8.012)

**Table 3:** Sensitivity, specificity, and predictive values of history, clinical, and laboratory parameters TLC, total leucocyte count; CRP, C-reactive protein

Parameters	Sensitivity (%)	Specificity (%)	Positive predictive value (%)	Negative predictive value (%)
Duration of fever (>4 days)	65.7	67	56.4	75
Rising trend of fever	41	91	75	70
Loose stool	30	83	53.7	64.6
Coated tongue	53.4	83.9	68.4	73.4
Splenomegaly	42.5	92.9	79.5	71.2
Hepatomegaly	47.9	13.4	26.5	28.3
TLC (4000-11,000/mm^3^)	83.56	50	52.14	82.35
Neutrophils (>50%)	87.67	25	43.24	75.68
Eosinophils (0%)	90.41	30.36	45.83	82.93
CRP (>25 mg/L)	82.19	53.57	53.57	82.19

Logistic regression identified several independent risk factors for EF, viz. duration of fever >4 days, rising trend of fever, coated tongue, splenomegaly, elevated CRP, elevated SGPT, and absolute eosinopenia, as shown in Table 2. Despite significant p-values, none of these parameters are specific enough to make a diagnosis of EF on an individual basis. However, the combined specificities of all the statistically significant lab parameters amount to 92.2%. When we add the statistically significant history and clinical parameters to this lab parameter group, the combined specificity further increases to 94.1%. This combined specificity of 94.1% allows us to confidently rule in the diagnosis of EF unless proved otherwise.

Other features included abdominal pain (27.4%), headache (11%), appetite loss (70%), constipation (4%), cough (18%), hemoglobin below 9 g/dL in 85% of cases, and erythrocyte sedimentation rate (ESR) above 10 mm/hr in 45%. However, these findings were not statistically significant.

## Discussion

This study identifies parameters for the early diagnosis of EF using simple history, clinical, and laboratory data in India. We found the highest incidence in the one-to-five-year age group, with a median age of 6.36 years. This aligns with Kumari et al. [5], who reported 94% of cases in children older than three years, and Walia et al. [6], who noted predominance in the 5-12 year age range. Three cases were below one year of age in our study. However, a study from Nigeria by Mulligan reported the highest incidence in the 10-19 year age group [7].

Both EF and NEF groups showed male predominance, consistent with other studies [5,8], showing a male-to-female ratio of 2.18 in Group I. The higher incidence in boys may be linked to increased outdoor activity and exposure to infection sources. In our study, most cases occurred around the monsoon season, which correlates with many other studies [8,9]. This discrepancy may be due to differences in the contaminated water supply during the monsoon season. Our study found that drinking RO-purified water minimized the likelihood of waterborne infections during rains. Group I exhibited a significantly higher prevalence of specific clinical features: fever duration exceeding four days (56.5%), a step-ladder pattern of rising fever (41%), loose stools (30%), coated tongue (53.4%), hepatomegaly (48%), and splenomegaly (42.5%). These findings are compared with existing literature in Table 4.

**Table 4:** Comparison of history, clinical, and laboratory parameters with other studies TLC, total leucocyte count; CRP, C-reactive protein; SGPT, serum glutamic pyruvic transaminase; EF, enteric fever

Parameters	Our study	Kumari et al. [5]	Nusrat et al. [10]	Behera et al. [11]
Study type	Prospective	Cross sectional	Prospective	Retrospective
Study duration	September 1, 2015 to February 28, 2017	March 2018 to April 2019	January 2017 to December 2019	January 2017 to December 2019
Total no. of cases included in the study	350	100	200	112
Diagnosis of EF	Blood culture +	Blood culture +	Blood culture +, Widal test +	Blood culture +, Widal test +, Typhidot +
Total no. of EF cases	73 EF cases (culture positive) out of a total of 350 febrile cases	75 EF (culture positive) cases out of a total of 100 febrile cases	200	112
Age group	6 month-15 years	<12 years	1-15 years	1-14 years
Fever + in EF cases included	73 (100%) with <1 week of fever	75 (100%) with >5 days of fever	200 (100%) with >5 days of fever	110 (98.2%)
Duration of fever calculated (significant) >4 days	48 (56.5%)	-	-	-
Rising trend of fever	30 (41%)	-	-	-
Diarrhea	22 (30%)	39 (52%)	33 (16.5%)	30 (26.7%)
Coated tongue	39 (53.4%)	32 (42.7%)	71 (35.5%)	2 (1.79%)
Splenomegaly	31 (42.5%)	-	32 (16%)	10 (8.93%)
Hepatomegaly	35 (48%)	32 (42.7%)	67 (34%)	18 (16.07%)
TLC count in normal range	61 (83.5%)	54 (72%)	149 (74.5%)	-
Leucocytosis	9 (12.3%)	4 (5.3%)	36 (18%)	22 (19.64%)
Leucopenia	3 (4.1%)	17 (22.7%)	15 (7.5%)	22 (10.71%)
Relative neutrophilia (>50%)	64 (87.7%)	-	66 (33%)	-
Absolute eosinopenia (with 0 counts)	66 (90.4%)	39 (52%)	-	66 (58.93%)
Raised CRP (>25 mg/L)	60 (82.2%)	-	-	82 (73.21%)
Raised SGPT	42 (57.5%) (with cut off >40 IU/L)	-	-	34 (30.36%)

Notably, we found no studies specifically examining the significance of fever duration in diagnosing EF in children. While fever is present in nearly all EF cases, our data suggest that a duration exceeding four days without focus may be a significant diagnostic indicator in endemic regions, as determined via ROC curve analysis. In our cohort of 73 confirmed cases, 56.5% had fever lasting longer than four days.

We also observed a rising trend of fever, characterized by a step-ladder pattern, in 41% of cases. Although traditionally associated with adults, to our knowledge, no studies have documented this pattern in pediatric populations.

Additional clinical features included loss of appetite (70%), cough (18%), abdominal pain (27.4%), constipation (4%), and headache (11%), highlighting symptom variability that may aid in diagnosis. Loose stools were present in 30% of cases, consistent with findings from other studies [5,11]. Although this was significantly higher compared to the NEF group, it did not emerge as an independent predictor in logistic regression analysis. Tongue examination revealed a yellowish or white coating, typically occurring after four days of fever. We recorded this in 53.4% of children with EF, a prevalence higher than splenomegaly and hepatomegaly, and identified it as an independent predictor in logistic regression analysis, comparable with other studies [5,9,10]. Bacteremia can lead to organ seeding, resulting in signs like hepatomegaly and splenomegaly. In our cohort, both hepatomegaly and splenomegaly were significant indicators of EF, with splenomegaly identified as an independent predictor, corroborating findings from other studies [10-13].

Regarding laboratory findings, we compared TLC, neutrophilia, eosinopenia, elevated CRP, and SGPT levels with existing literature, finding consistent results [5,10,11]. In this study, normal TLC was identified in 83.5% (61 out of 73) cases in Group I. Out of these, 54 cases were *S.* Typhi and seven cases were *S.* Paratyphi infections. Leukocytosis was found in nine cases (12.3%), of which eight cases were seen in *S. *Typhi infections and one in *S.* Paratyphi infection. We observed leucopenia in three cases (4.1%) in Group I, all of which had *S.* Typhi infection. We also looked at relative neutrophilia (neutrophils >50%) in our study. Notably, 87.7% of cases had relative neutrophilia. On further breakdown, 88.5% of the normal TLC group (47 cases of *S.* Typhi and seven cases of *S.* Paratyphi) had relative neutrophilia. In addition, 77.7% of leukocytosis cases (seven of the nine cases) had relative neutrophilia. All cases of leucopenia (three cases) had relative neutrophilia. Neutrophilia was found in only 33% of cases by Nusrat et al. [10] in their study. In our study, we identified relative neutrophilia as a significant and independent predictor for EF, which has been studied by Nusrat et al. to the best of our knowledge.

Eosinophils can be influenced by adrenal glucocorticoids and epinephrine. The absolute eosinopenia in our EF group likely results from the rapid sequestration of eosinophils due to chemotactic factors such as C5a and fibrin, which are present in early infection [14,15]. Our findings align with other studies [5,11,16], although some reported lower rates of eosinopenia due to high baseline eosinophil counts from endemic parasitic infections or pollutants [12,17].

CRP serves as a marker for bacterial sepsis. In our study, elevated CRP emerged as a strong independent predictor of EF, positive in 82.2% of EF cases, comparable to the 73.21% reported by Behera et al. [11].

Transaminitis was found in 57.5% of EF cases, while Chitkara et al. reported a 77% incidence in Delhi [18]. Other studies reported lower transaminitis rates, which can be attributed to higher cutoffs used for diagnosis [17,19].

In our study, the combined specificity of these statistically significant history, clinical, and lab parameters resulted in a very high specificity of 94.1%. This combined specificity of 94.1% definitely allows us to rule in the diagnosis of EF unless proved otherwise. To the best of our knowledge, this kind of clinical and basic laboratory assessment to predict EF has not been published by any author.

Limitations

A hospital-based study over a limited period might not reflect the true picture of the population. A larger sample size would have been better for more accurate results. In our study, information regarding prior use of antibiotics was not known. Even though blood culture sensitivity is high despite prior antimicrobial use, some children might have been excluded from the analysis due to negative culture results.

## Conclusions

Based on our findings, we propose that simple investigations, combined with clinical assessments such as a fever duration exceeding four days, a rising trend of fever, coated tongue, splenomegaly, elevated CRP levels (>25 mg/L), elevated SGPT levels (>40 IU/L), and eosinopenia (0%), can serve as strong predictors for the early diagnosis of EF. This approach may not only help reduce unnecessary antimicrobial use in children who do not meet these diagnostic criteria, but also timely identify EF patients for the appropriate treatment available.
